# COX-1/PGE_2_/EP4 alleviates mucosal injury by upregulating β-arr1-mediated Akt signaling in colitis

**DOI:** 10.1038/s41598-017-01169-6

**Published:** 2017-04-21

**Authors:** Xiaojie Peng, Jianzhong Li, Siwei Tan, Minyi Xu, Jin Tao, Jie Jiang, Huiling Liu, Bin Wu

**Affiliations:** Department of Gastroenterology, The Third Affiliated Hospital of Sun Yat-Sen University, Guangzhou, China

## Abstract

COX-1/PGE_2_ is an important protective mediator in ulcerative colitis (UC). β-arrestin1 (β-arr1), which acts as a scaffold protein, is involved in PGE_2_-mediated signaling pathways. However, the interaction between PGE_2_ and β-arr1 in maintaining mucosal barrier integrity remains unexplored. In this study, we demonstrated that COX-1 and PGE_2_ were significantly decreased, and *EP4* mRNA was downregulated in both UC patients and mice during the injury phase. PGE_2_ treatment was found to alleviate mucosal injury and induce EP4 expression during dextran sulfate sodium (DSS)-induced colitis in wild-type (WT) mice. Following DSS-induced injury, *β-arr1* deficient mice showed increased signs of colitis compared to *β-arr1* WT mice, and the expression of PI3K and p-Akt were remarkably downregulated in *β-arr1* deficient mice. In parallel, HCT116 cells transfected with *β-arr1* siRNA were examined in the presence or absence of PGE_2_ *in vitro*. PGE_2_ treatment in the *β-arr1* WT/KO DSS model and *β-arr1* siRNA transfection of HCT116 cells confirmed that PGE_2_ upregulated β-arr1 *in vivo* and *in vitro*. Collectively, our results indicate that COX-1/PGE_2_/EP4 upregulates the β-arr1 mediated Akt signaling pathway to provide mucosal protection in colitis. Thus, these findings provide support for the future development and clinical application of COX-1/PGE_2_ in UC.

## Introduction

Inflammatory bowel disease (IBD), a multifactorial disease perpetuated by a dysregulated immune response, includes ulcerative colitis and Crohn’s disease. The etiology of IBD involves a complex interaction of genetic predisposition, environmental triggers, microbial factors and immune responses^1^. Ulcerative colitis is a chronic, idiopathic, and inflammatory disease of the rectal and colonic mucosa. The incidence and prevalence of UC are increasing worldwide^2^. No innovative treatment has been developed, although progress has been made in the overall management of the disease. Conventional nonsteroidal anti-inflammatory drugs (NSAIDs) are occasionally involved in the development of de novo colitis, although they are more frequently implicated in the aggravation of pre-existing intestinal diseases^3^. This is most obviously demonstrated in patients with inflammatory bowel disease, who frequently require anti-inflammatory analgesics due to peripheral arthritis, sacroiliitis, ankylosing spondylitis and osteoporosis-related fractures^4^. The administration of NSAIDs is one of the major risk factors in triggering the clinical relapse of IBD^5^. Cyclooxygenase (COX) has two isoforms, COX-1 and COX-2^6^. COX-1 is expressed constitutively in epithelial cells in the crypt with the exception of the villi and it has been proposed to maintain the cell integrity of the gastrointestinal tract^7^. In the normal intestine, COX-2 is not expressed in appreciable amounts in epithelial cells but is expressed in colonic adenomas, carcinomas and IBD^8–10^. Cyclooxygenase catalyzes the production of prostanoids, which are a group of eicosanoids consisting of four types of prostaglandins (PGs) and thromboxanes: PGE_2_, PGD_2_, PGF_2α_, PGI_2_ and TXA_2_. The PGE_2_ receptor is EP^11^. EP consists of four subtypes: EP1, EP2, EP3 and EP4. All of these subtypes respond to a naturally occurring agonist, PGE_2_, but differ in their actions and responses to various analogues^12^. The EP4 receptor has been shown to stimulate cAMP/protein kinase A (PKA) signaling through the activation of G_s_α and adenylate cyclase in the regulation of growth and proliferation^13^. Furthermore, EP4 also elicits the activation of phosphoinositide3-kinase (PI3K) via the β-arrestin (β-arr) pathway^14^.

β-arr1 and β-arr2, two of the four members of the arrestin family, are scaffolding proteins that modulate GPCR signaling pathways and signal transduction. β-arrs are involved in several types of diseases, including Parkinson’s disease, multiple sclerosis, cardiovascular conditions, and colitis by serving as a scaffold protein in various signaling pathways, including ERK1/2, Wnt/β-catenin and c-Src^15–17^. Previously, we discovered that β-arr2 contributed to the development of experimental colitis and mucosal repair in the process of UC^18, 19^. In addition to their role in receptor downregulation, β-arrs also act as scaffold proteins to accelerate signal transduction in the EP4 pathway. The physical association between EP4 and β-arr1 was demonstrated using bioluminescence resonance energy transfer^20^. β-arrs also have been reported to play a role in cell proliferation, apoptosis and differentiation via several signaling pathways^21^. Additional studies have shown that β-arr1 was involved in the activation of the PI3K/Akt pathway and affects apoptosis or cell survival^22^.

In the present study, we found that PGE_2_ and the PGE_2_ receptor, EP4, were downregulated in both ulcerative colitis patients and mice with DSS-induced experimental colitis. In addition, treatment with PGE_2_ alleviated mucosal injury in colitis. We also found that expression of β-arr1 decreased in both human and mice colitis. Targeted deletion of *β-arr1* increased signs of colitis compared to *β-arr1* wildtype (WT) mice following DSS-induced injury by activating PI3K/Akt signaling. Moreover, PGE_2_ contributed to the preservation of epithelial proliferation of experimental colitis by mainly enhancing EP4/β-arr1/p-Akt signaling. Taken together, these findings demonstrated the pivotal role of β-arr1 in the integrity of the PGE_2_-mediated colonic epithelial barrier and provided sufficient scientific evidence to establish EP4/β-arr1/p-Akt signaling as a new therapeutic target of UC.

## Results

### Expression of COX, prostaglandins and prostaglandin receptors in colitis

**Figure 1:**
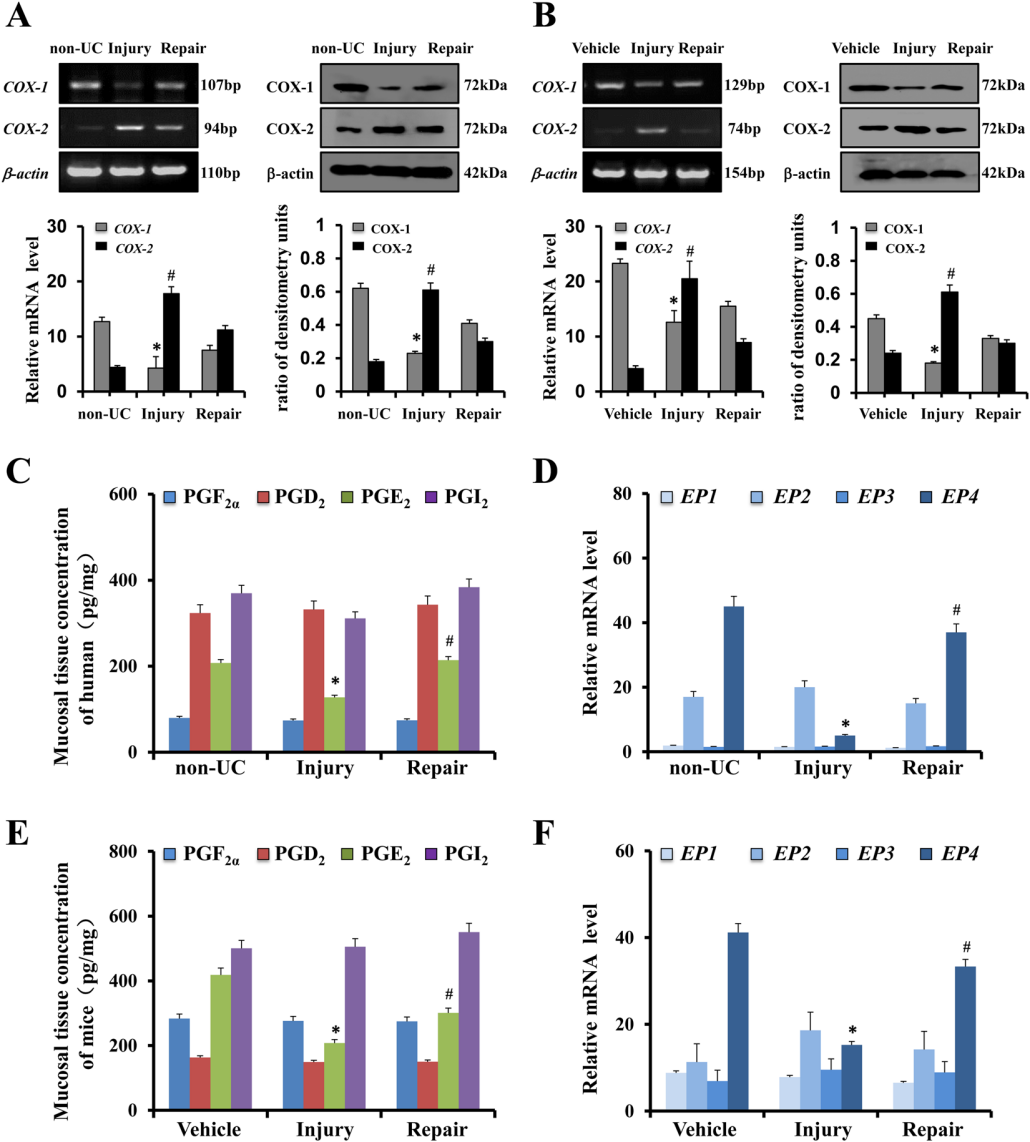
Expression of COX, prostaglandins and prostaglandin receptors in colitis. (**A**) COX-1 and COX-2 expression in colon tissues were determined using RT-PCR and Western blotting in the non-UC group (human normal colon tissue), injury group (colitis colon tissue in the injury phase) and repair group (colitis colon tissue in the repair phase); β-actin was used as a loading control (n = 6 per group). *P < 0.05, ^#^ *P* < 0.05 non-UC versus injury. (**B**) COX-1 and COX-2 expression in mouse colon tissue was determined using RT-PCR and Western blotting analyses in the vehicle group, injury group and repair group. (n = 4 per group) *P < 0.05, ^#^ *P* < 0.05 vehicle versus injury (**C**) Concentrations of mucosal prostaglandins PGE_2_, PGD_2_, PGF_2α_ and PGI_2_ in of three pairs of representative human specimens. Values are expressed as the mean ± SD (n = 6 per group). *P < 0.05, non-UC versus injury phase. ^#^ *P* < 0.05 injury phase versus repair phase (**D**) mRNA expression of *EP1*, *EP2*, *EP3* and *EP4* in the colonic mucosa were evaluated using real-time PCR in the indicated group. Values are expressed as the mean ± SD (n = 6 per group). *P < 0.05, non-UC versus injury phase. ^#^ *P* < 0.05 injury phase versus repair phase (**E**) Concentrations of mucosal prostaglandins PGE_2_, PGD_2_, PGF_2α_ and PGI_2_ in the mouse vehicle group, injury group and repair group; The values are expressed as the mean ± SD (n = 4 in each group). *P < 0.05 versus vehicle mice, ^#^ *P* < 0.05 injury group versus repair group. (**F**) *EP1*, *EP2*, *EP3* and *EP4* mRNA expression in the colonic mucosa of mice were evaluated by real-time PCR in the indicated group. The values are expressed as the mean ± SD (n = 4 in each group). *P < 0.05 versus vehicle mice, ^#^ *P* < 0.05 injury group versus repair group.

To examine the role of COX in UC, colon mucosal specimens from colitis patients and healthy volunteers were analyzed. The expression pattern of COX-1 mRNA was markedly suppressed in UC patients, whereas COX-2 was significantly increased in the patients’ mucosa in the injury phase. Western blotting revealed that colonic specimens obtained from patients with UC displayed reduced COX-1 protein expression, whereas the expression of COX-2 protein was increased (Fig. 1A). Similar results were found in animal experiments, in which COX-1 mRNA levels and protein levels decreased in DSS-treated mice. When DSS was withdrawn, the expression of COX-1 nearly returned to the levels observed in untreated controls (Fig. 1B). To further investigate the expression of prostaglandins in colitis, colon mucosal specimens from colitis patients and healthy volunteers were analyzed. As shown in Fig. 1C, the concentrations of PGE_2_, PGD_2_, PGF_2α_, and PGI_2_ were measured in biopsies of rectal mucosa using an ELISA kit. The PGE_2_ concentration in the control group was 207.27 ± 6.8 pg/mg of protein, while PGE_2_ concentrations of the patients’ mucosa in the injury phase revealed decreased concentrations (127.38 ± 4.9 pg/mg of protein), and these differences were significant (*p* < 0.05). In contrast, the PGE_2_ concentration (213.78 ± 8.7 pg/mg of protein) of the patients’ mucosa in the repaired phase showed significant differences compared with the in the injury phase. No significant changes in the expression of other prostaglandins were observed (Fig. 1C). To further investigate the expression of PGE receptors, *EP1, EP2, EP3* and *EP4* mRNA levels were analyzed using real-time PCR in human normal colon tissue and colitis colon tissue (injury and repaired phases). There were no apparent differences in the levels of *EP1, EP2*, and *EP3* mRNA between the normal colon tissue and the colitis colon tissue (injury and repaired phases), but a significant difference in the level of *EP4* was observed (Fig. 1D). Furthermore, *EP4* mRNA revealed a decrease in colitis during the injury phase. To further study the role of prostaglandins and receptors in UC, DSS was used to induce colitis in mice, and similar results were found in animal experiments (Fig. 1E,F).

### PGE_2_/EP4 alleviates mucosal injury in colitis

**Figure 2:**
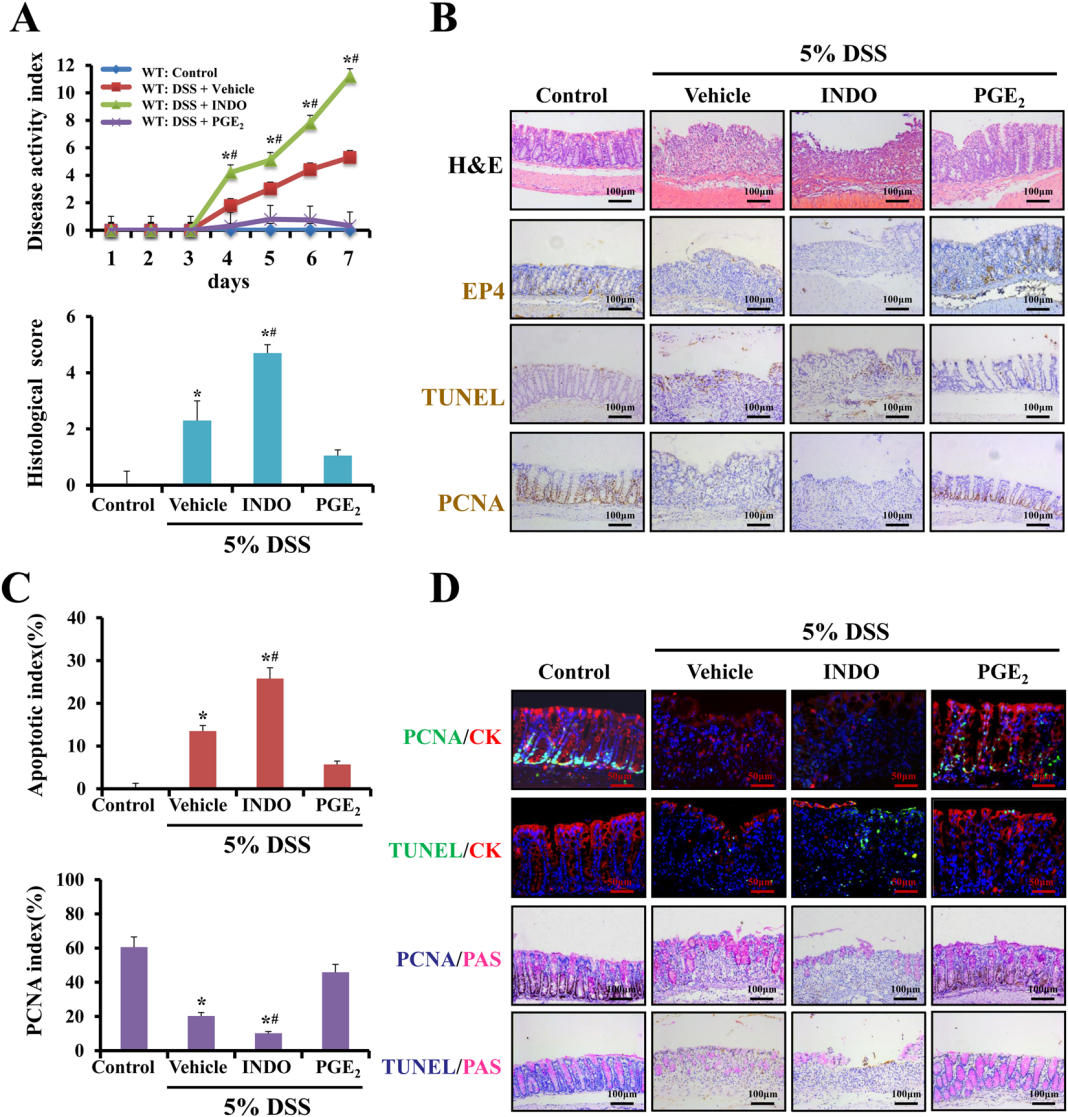
PGE_2_/EP4 alleviates mucosal injury in colitis. (**A**) The disease activity index was determined at the indicated time points as described in the Methods. Histological damage after DSS treatment for 7 days was scored after H&E staining as described in the Methods. *P < 0.05 compared with control group mice. ^**#**^ *P* < 0.05 versus vehicle group (n = 4 in each group). (**B**) Representative photomicrographs of H&E staining, TUNEL staining (brown, ×200), immunostaining of EP4 (brown, ×200) and PCNA (brown, ×200) in colonic sections of WT littermates in the indicated group. (n = 4 in each group). (**C**) The apoptotic index was measured by quantifying TUNEL signals in 100 random fields per section. The percentage of PCNA-positive cells is represented graphically. Values are expressed as the mean ± SD. *n* = 6 in each group, ^*^ *P* < 0.05 versus control mice, ^**#**^ *P* < 0.05 versus vehicle group. (**D**) Double stain for PAS and PCNA and PAS and TUNEL in four groups. PAS for goblet cells is pink (×200). Immunostaining of PCNA and TUNEL are shown in brown. Double immunofluorescence stain for cytokeratin and PCNA, cytokeratin and TUNEL in the indicated group (×400).Nuclei are stained with DAPI in blue. Localization of PCNA and TUNEL are visualized in green and cytokeratin is stained in red. The merging positive signals of PCNA or TUNEL and cytokeratin are visualized in yellow. (n = 4 in each group).

To examine whether PGE_2_/EP4 signaling contributed to injury in colitis, we employed colitis mouse models. Mice were randomly divided into the control group, UC model group, indomethacin group (DSS treatment administered with indomethacin) and PGE_2_ group (DSS treatment administered with PGE_2_). The macroscopic finding of the mice treated with 5% DSS and sacrificed on day 7 showed that the intestines of the mice had edema and hemorrhagic redness all throughout the colon and cecum (Supplementary Fig. S1). In response to DSS treatment, mice displayed features of colitis characterized by a loss in body weight, loose stools and occult blood in the feces. Colonic mucosa suffered from crypt destruction, goblet cell loss and inflammatory cell infiltration. The indomethacin group developed worsened symptoms while the PGE_2_ group exhibited clinical symptoms, such as diarrhea and an attenuation of hemoccult. Consequently, PGE_2_ significantly suppressed the histological injury and the disease activity index scores (Fig. 2A). Immunostaining showed that EP4 and PCNA were increased in mice in the PGE_2_ group (Fig. 2B). Quantification of PCNA-positive cells demonstrated that the proliferation index was higher in the PGE_2_ group. Furthermore, epithelial apoptosis, which was stained by TUNEL, was significantly reduced in the PGE_2_ group (Fig. 2C). Expectedly, double labeling of cytokeratin and PCNA, PAS and PCNA revealed that PGE_2_ increased the regeneration of epithelial cells and goblet cells. However, double staining of cytokeratin and TUNEL, and PAS and TUNEL demonstrated that indomethacin attenuated the regeneration of epithelial cells and goblet cells (Fig. 2D). Taken together, these findings further confirm that PGE_2_ alleviates mucosal injury in colitis.

### β-arr1 is downregulated in active colitis

**Figure 3:**
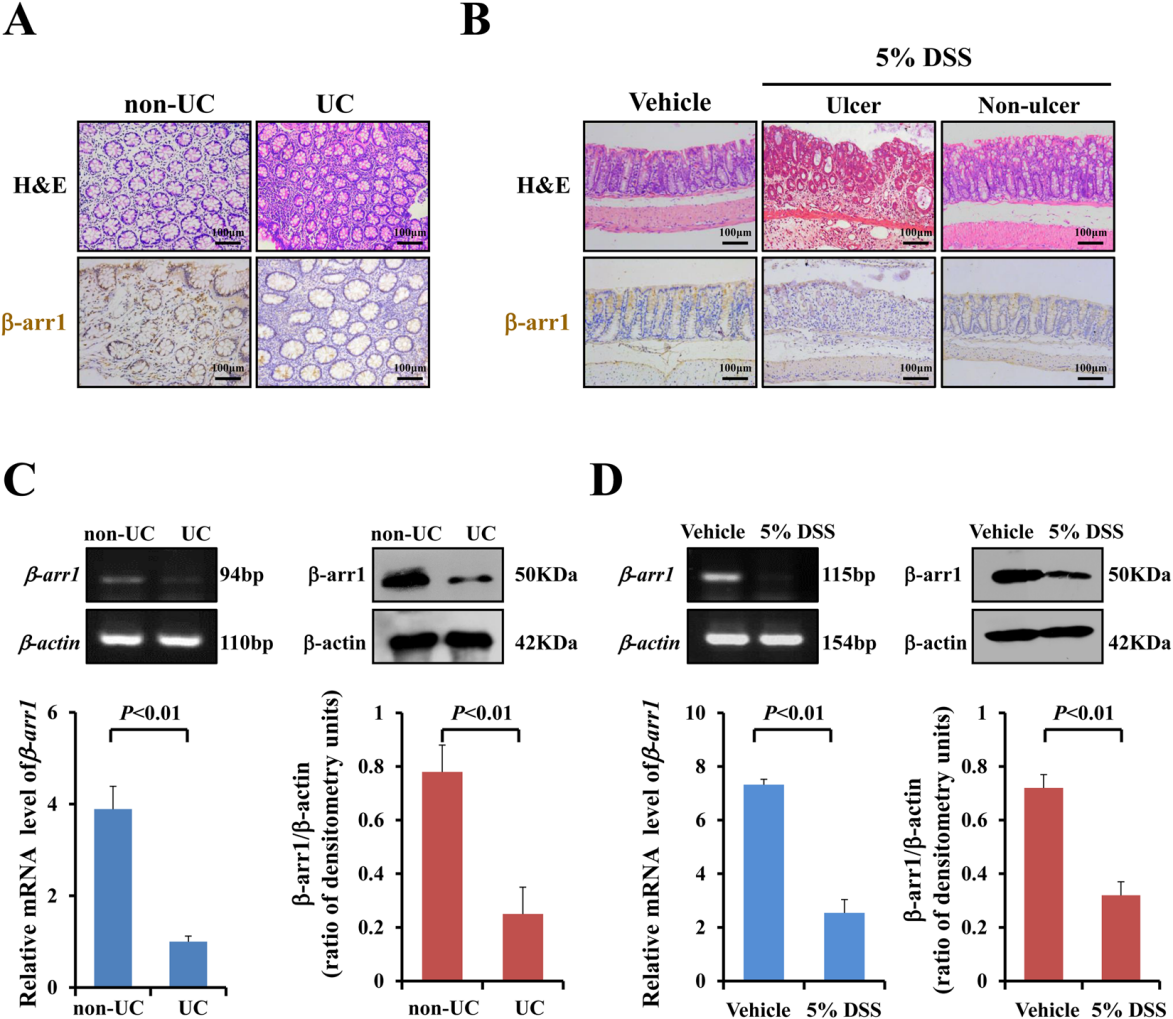
β-arr1 is downregulated in active colitis. (**A**) Immunostaining of β-arr1 in human colonic mucosa in the healthy volunteer group and UC group (brown, ×200). (n = 4 in each group). (**B**) Immunostaining of β-arr1 in mouse colonic mucosa in the control group, and ulcer sections and non-ulcer sections in the DSS group (brown, ×200). (n = 6 in each group). (**C**) The expression of intestinal mucosal β-arr1 mRNA and protein was evaluated in human colonic mucosa in the healthy volunteer group and UC group using real-time PCR and western blotting. Values are expressed as the mean ± SD. (n = 6 in each group). *P < 0.01 versus control group. (**D**) The expression of intestinal mucosal β-arr1 mRNA and protein was evaluated in the mouse colonic mucosa in the control group and DSS group using real-time PCR and western blotting. Values are expressed as the mean ± SD. (n = 6 in each group). *P < 0.01 versus vehicle mice. DSS: dextran sulfate sodium; Non-UC: healthy volunteers; UC: ulcerative colitis.

Immunohistochemistry studies were performed to confirm β-arr1 expression in normal colon tissues and colitis tissues. Moreover, β-arr1 expression was decreased in colonic specimens in UC patients (Fig. 3A). At both the mRNA and protein levels, β-arr1 expression was examined in normal colon tissues and colitis colon tissues. These results showed that β-arr1 expression was significantly downregulated in colitis colon tissue compared with normal colon tissues (Fig. 3C). To further study the role of β-arr1 in UC, DSS was used to induce colitis in mice. Interestingly, both β-arr1 mRNA and protein levels decreased in the DSS-induced intestinal mucosa (Fig. 3D). Simultaneously, immunostaining results of β-arr1 showed little positive signal in the ulceration area compared with the vehicle. However, there were more signals in the non-ulcerative area compared with the ulcerative area (Fig. 3B). These results suggest that β-arr1is downregulated in colitis in both humans and mice.

### Targeted deletion of *β-arr1* exacerbates DSS-induced colitis in mice

**Figure 4:**
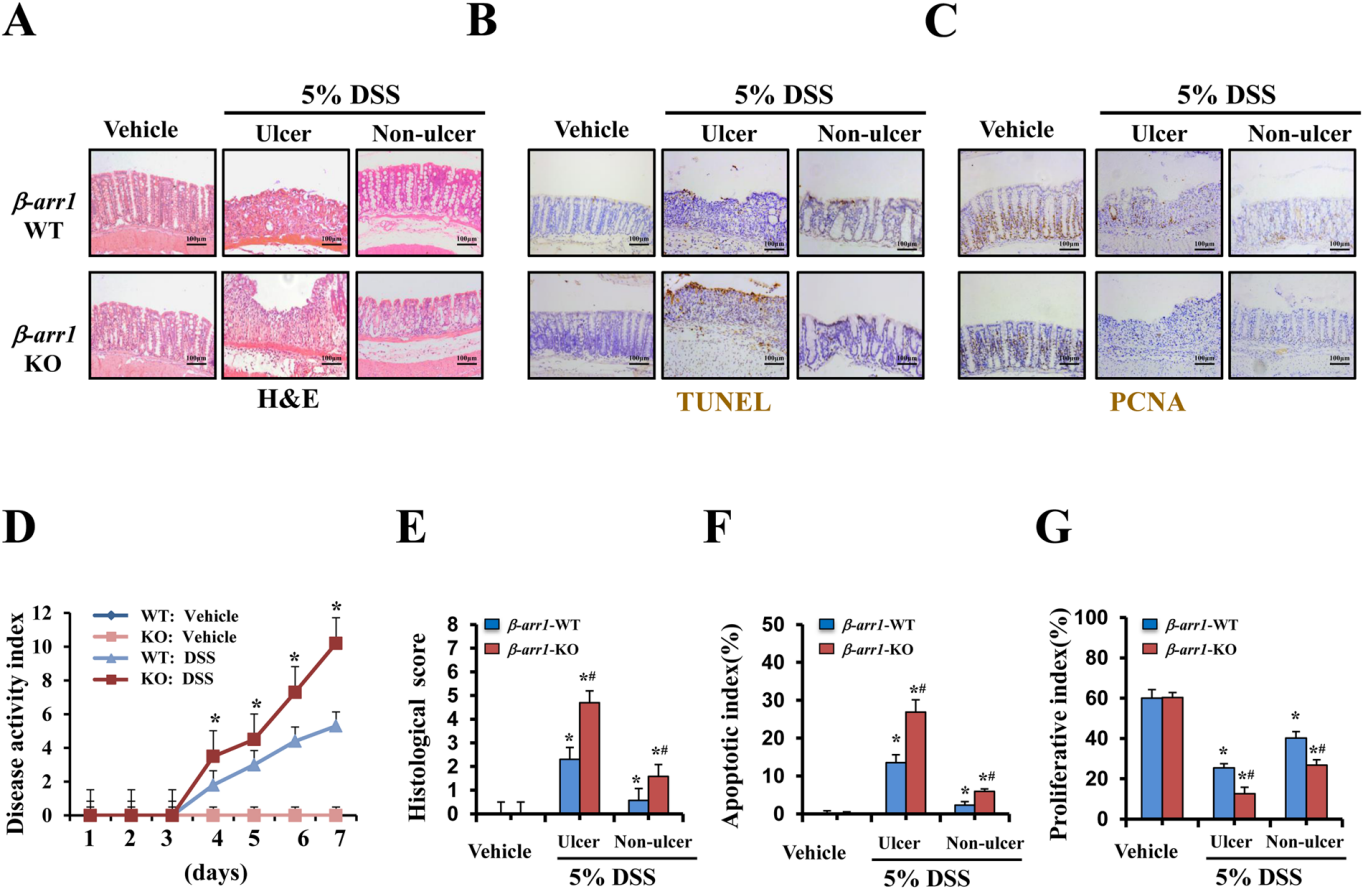
Targeted deletion of *β-arr1* exacerbates DSS induced colitis in mice. (**A**) Representative photomicrographs of H&E staining in colonic sections of *β-arr1* WT and KO mice in the control group and the ulcer section and non-ulcer section of the DSS group (×200, n = 4 per group). (**B**) TUNEL staining revealed apoptotic induction in intestinal epithelial cells of *β-arr1* WT and KO mice (brown, ×200, n = 4 per group). (**C**) Immunohistochemical staining for PCNA in colonic sections of *β-arr1* WT and KO mice in the control group and the ulcer section and non-ulcer section of the DSS group. (brown, ×200, n = 4 per group). (**D**) The disease activity index of mice treated with or without DSS of WT and *β-arr1* KO mice was measured at the indicated time points. *P < 0.01 compared with the WT mice (n = 4 per group). (**E**) Histological damage in colonic tissues obtained from the mice treated with DSS or without DSS of *β-arr1* WT and KO mice was scored after H&E staining. *P < 0.05 compared with the control mice, ^#^ *P* < 0.05, *β-arr1* WT mice versus KO mice. n = 6 per group. (**F**) Apoptotic index was measured by quantifying TUNEL signals in 100 random fields per section. Values are expressed as the mean ± SD. *n* = 6 in each group, *P < 0.05 compared with the control mice, ^#^ *P* < 0.05, *β-arr1* WT mice versus KO mice. (**G**) The percentage of PCNA-positive cells is represented graphically. Values are expressed as the mean ± SD. *n* = 6 in each group, *P < 0.05 compared with the control mice, ^#^ *P* < 0.05, *β-arr1* WT mice versus KO mice.

The above observations prompted us to use *β-arr1*-gene-deficient models to further investigate the role of β-arr1 in colitis. To assess the extent and severity of colonic pathological changes, we measured the length of colons from mice subjected and not subjected to colitis. Although DSS-induced colitis resulted in colon shortening in both *β-arr1* WT and KO mice, the colon was still significantly longer in *β-arr1* WT mice than in the KO mice (Supplementary Fig. S2). Disease activity index scores were significantly lower in *β-arr1* WT mice compared with KO mice (Fig. 4D). Fewer and smaller colonic ulcers were also detected in *β-arr1* WT mice compared with KO mice after DSS treatment (Supplementary Fig. S2). Consistent with the overall phenotypic differences in ulcer status, clinical signs and total colon morphology, H&E-stained microscopic sections of the colon revealed marked differences between *β-arr1* WT mice and KO mice. In addition, histological analysis revealed substantially less epithelial damage and disruption of crypt architecture in *β-arr1* WT mice (Fig. 4A,E). Simultaneously, immunostaining showed highly positive TUNEL signals in *β-arr1* KO mice after DSS treatment (Fig. 4B,F). Cytokeratin and PAS immunostaining indicated that targeted deletion of *β-arr1* exacerbated the regeneration of epithelial cells and goblet cells (Supplementary Fig. S2). Immunostaining studies also showed that the expression of PCNA decreased during colitis periods, and *β-arr1* KO mice exhibited significantly decreased levels compared with WT mice (Fig. 4C,G). These results reveal that the critical role of β-arr1 in colitis is associated with epithelial cell apoptosis.

### β-arr1 activates PI3K/Akt signaling in colitis

**Figure 5:**
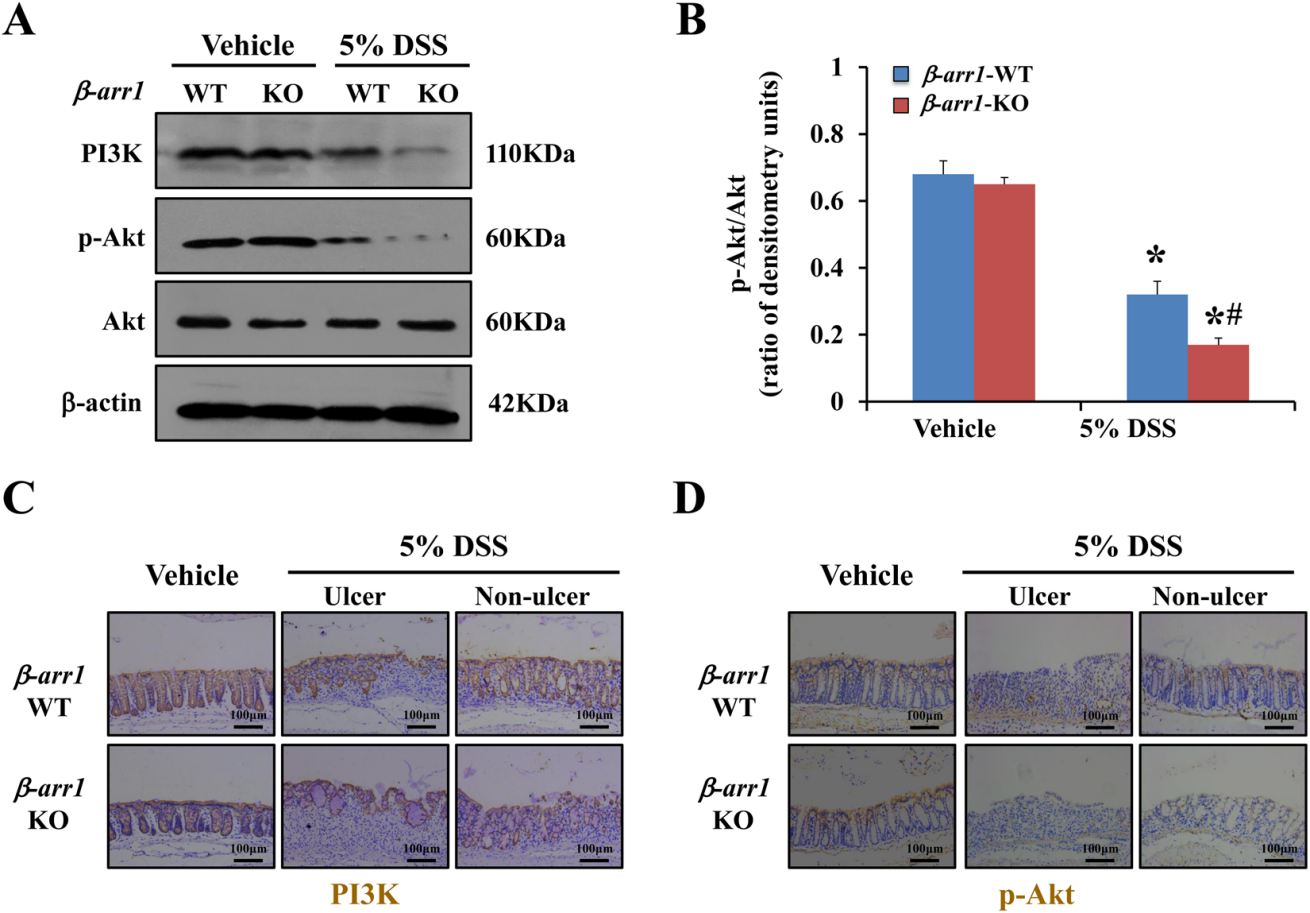
β-arr1 actives PI3K/Akt signaling in colitis. (**A**) Western blot of PI3K, p-Akt and Akt in colonic mucosa of *β-arr1* WT and KO mice with or without DSS treatment at the indicated time points. All western blot images are representative images from five independent experiments, and β-actin was used as a loading control. (**B**) Quantitative analysis of p-Akt/Akt as measured by densitometry scanning of Western blots. Values are expressed as the mean ± SD of five independent experiments, *P < 0.05 versus vehicle group, ^*#*^ *P* < 0.05 versus *β-arr1* KO mice after 5% DSS treatment. (**C**) Immunohistochemical staining for PI3K in colonic sections of *β-arr1* WT and KO mice in the control group, and the ulcer section and non-ulcer section in the DSS group (brown, ×200, n = 4 per group). (**D**) Immunohistochemical staining for p-Akt in colonic sections of *β-arr1* WT and KO mice in the control group, and the ulcer section and non-ulcer section in the DSS group (brown, ×200, n = 4 per group).

Previous reports have shown that β-arr1 is an important signaling scaffold that facilitates the activation of numerous effector pathways regulating cellular proliferation, differentiation, and apoptosis, such as the MAPK and PI3K signaling pathways. The PI3K-Akt pathway plays an important role in many diseases^15^. To confirm whether PI3K/Akt is targeted downstream by β-arr1 in colitis, we used *β-arr1* WT and *β-arr1* KO mice to induce colitis via DSS. Western blots showed that PI3K and Akt phosphorylation were remarkably downregulated in DSS-induced colitis in *β-arr1* WT and KO mice compared with the control group. However, this decrease was markedly exacerbated in *β-arr1* KO mice compared with WT mice (Fig. 5A,B). Similar results were also observed in immunohistochemical staining (Fig. 5C,D). These results suggest that β-arr1 activates PI3K/Akt signaling in colitis.

### PGE_2_/EP4 upregulats β-arr1 mediated Akt signaling *in vivo* and *in vitro*

**Figure 6:**
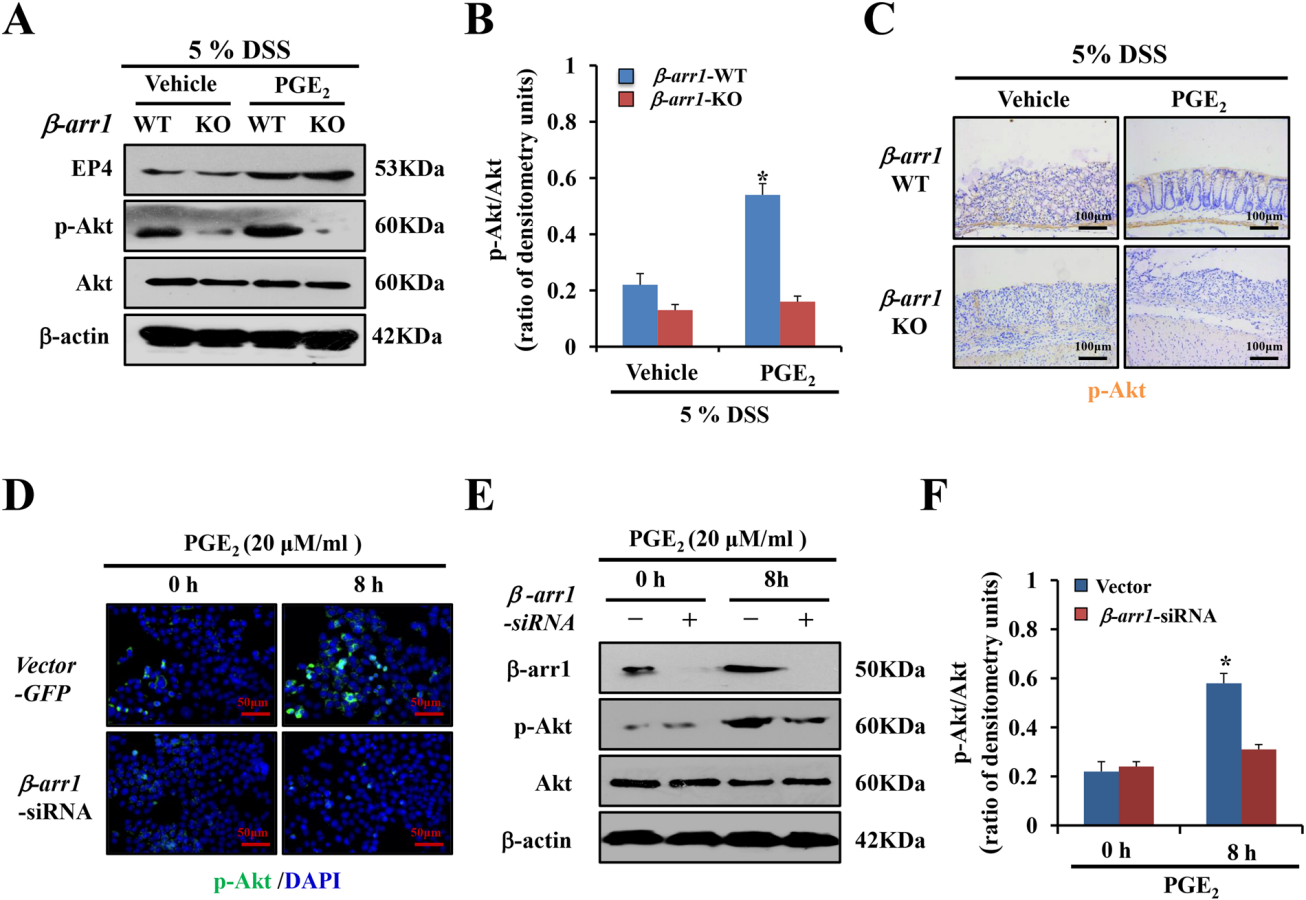
PGE_2_/EP4 upregulates β-arr1 mediated Akt signaling *in vivo* and *in vitro*. (**A**) Western blotting of EP4, p-Akt and Akt in colonic mucosa of *β-arr1* WT and KO mice treated with DSS with or without PGE_2_ at the indicated time points. All western blot images are representative images from three independent experiments, and β-actin was used as a loading control. (**B**) Quantitative analysis of p-Akt/Akt ratio as measured by densitometry scanning of Western blots. Values are expressed as the mean ± SD of three separate experiments, *P < 0.05 versus vehicle. (**C**) Immunohistochemical staining for p-Akt staining in colonic sections of *β-arr1* WT and KO littermates under DSS with or without PGE_2_ treatment at the indicated time points (brown, ×200, n = 4 per group). (**D**) Expression of p-Akt in HCT116 cells transfected with control or *β-arr1* siRNA after PGE_2_ treatment. Nuclei were stained with DAPI in blue. Localization of p-Akt was visualized in green. (**E**) HCT116 cells were stimulated with or without PGE_2_ (20 μm/ml) for 8 h in the presence of control siRNA or *β-arr1* specific siRNA. The lysates were subjected to Western blotting analysis for β-arr1, p-Akt and Akt. All western blot images are representative images from three independent experiments, and β-actin was used as a loading control. (**F**) Quantitative analysis of the p-Akt/Akt ratio as measured by densitometry scanning of Western blotting. Values are expressed as the mean ± SD of three experiments, *P < 0.05 versus control siRNA group.

Current views indicate that PGE_2_ promotes epithelial proliferation after mucosal injury, and we also know that targeted deletion of *β-arr1* exacerbates DSS-induced colitis in mice. Based on this knowledge, we investigated whether PGE_2_ could improve symptoms in mice with established colitis after DSS treatment. A western blot and immunohistochemical staining showed that Akt phosphorylation was remarkably upregulated in *β-arr1* WT mice with established colitis after PGE_2_ treatment. In contrast, there was no change in the expression of p-Akt in *β-arr1* KO mice after PGE_2_ treatment (Fig. 6A–C). However, in both *β-arr1* WT and KO mice, the expression of EP4 significantly increased during colitis periods after PGE_2_ treatment as detected by Western blot (Fig. 6A).

To further confirm these results, the effects of *β-arr1* siRNA on EP4 signaling were also examined. Transfection of *β-arr1* siRNA reduced the level of β-arr1 protein by 63% compared with that of non-targeting control siRNA. Similarly, EP4 expression was unaffected by β-arr1 downregulation. After *β-arr1* siRNA treatment, the expression of p-Akt was downregulated as shown by cell immunofluorescence and Western Blotting, indicating that silencing of *β-arr1* gene expression markedly attenuated the activation of p-Akt and suppressed cell proliferation (Fig. 6D–F). In summary, these data demonstrates that PGE_2_/EP4 upregulates β-arr1-mediated Akt signaling *in vivo* and *in vitro*.

## Discussion

PGE_2_ is present throughout the gastrointestinal tract and is known to elicit a variety of actions in the gut^23^. However, the role of PGE_2_ in the onset or alleviation of mucosal injury is not clearly known. In this study, we demonstrated that PGE_2_ treatment alleviated mucosal injury in colitis. We also revealed the role of EP4 receptors and PGE_2_-induced β-arr1/p-Akt signaling in maintaining epithelial barrier integrity.

PGs can protect the gastrointestinal (GI) tract^24, 25^. PGE_2_, the most abundant gastrointestinal prostaglandin, regulates many of the normal homeostatic functions of the gut, including motility and secretion^25^. The versatility of PGE_2_ is attributed to its differential local production and its selectivity for EP receptor subtypes (EP1, EP2, EP3 and EP4). Prostaglandins are synthesized through COX-1 and COX-2 and exhibit both pro- and anti-inflammatory effects. COX-1 maintains the integrity of the GI mucosa under physiological conditions, and COX-2 has been implicated in invasive events, such as inflammation^6, 7, 10^. In the present study, DSS treatment resulted in a loss of COX-1 expression in the crypt epithelium and an increase in COX-2 expression^10, 26^. A subsequent study showed that administration of a selective COX-2 inhibitor has no effect on either apoptosis or crypt survival; similarly, radiation of COX-1 deficient mice results in increased apoptosis and decreased crypt survival compared to WT mice^27^. These findings show that PGE_2_ produced through COX-1 plays an important role in epithelial homeostasis. The role of PGE_2_ in UC is still controversial. Previous study showed that COX-2 derived PGE_2_ played the pro-inflammatory role in UC^28, 29^. However, ours and other plenty of evidence showed that PGE_2_ release was suppressed in irritable bowel syndrome, radiation-induced small intestine injury, diverticular disease and so on^30–32^, and PGE_2_ protected the gastrointestinal tract through maintaining the mucosal integrity, limiting the inflammatory response and promoting the wound healing^25, 33, 34^. What is interesting, the local PGE_2_ level within tissues is difficult to measure due to the short half-life of PGE_2_ and the level of intestinal PGE_2_ changed dynamicly in mice experimental colitis^35–37^. Moreover, the reported contradictory mucosal PGE_2_ data may be due to the difference in the position of biopsy specimen, scored degree of mucosal injury and the phase of colitis in different researches^28, 29, 38^. In our study, we focused on the COX-1 derived-PGE_2_ in the epithelial cells and the biopsy samples were taken from the ulcer edge including the ulcerative tissue and the uninvolved tissue, while other researchers collected tissue samples from the most active inflammatory part of the colon to test the PGE_2_ as a surrogate marker^28^. But the cellular origins of these PGs and the relative contributions of COX-1 and COX-2 to their production have not been fully investigated and the precise underlying mechanism of these differences will be explored in our future study. Next, we found that PGE_2_ concentration decreased in the injury phase of human colitis, which was the only prostaglandin to show a significant change among the analyzed prostaglandins. In addition, there was a significant change in the *EP4* mRNA levels among the analyzed EP receptors. Current views indicate that the PGE_2_ pathway has a specific physiological significance in humans, which was highlighted by the finding that the *PTGER4* gene, which encodes the PGE_2_ receptor EP4, demonstrates a strong genetic association with both forms of IBD^39^. Zhang *et al*.^40^ discovered that inhibiting 15-hydroxyprostaglandin dehydrogenase, an enzyme that physiologically oxidizes PGE_2_ to prevent it from binding to prostaglandin receptors, led to improvements in hematopoietic stem cell transplants and colitis recovery during injury. The PGE_2_ level has dynamic change process in mice experimental colitis. In a study by Tessner, TG *et al*.^26^, PGE_2_ levels in cecal tissue were diminished after 5 days of DSS treatment, and PGE_2_ levels were dramatically increased during recovery from DSS-induced injury. Similar results were found in other reasearches^35–37^. Kandil *et al*.^41^ suggested that low endogenous levels of PGE_2_ predisposed relapses in a rat model of enterocolitis, and supported the anti-inflammatory activity of PGE_2_. A subsequent study showed that mPGES-1 deficient mice (PGES-1 is the terminal synthase for inducible PGE_2_ formation) had a greater susceptibility to acute DSS-induced injury, indicating a specific protective role for PGE_2_^42^. Taken together, these published studies corroborate our finding that COX-1/PGE_2_/EP4 contributes to the maintenance of mucosal integrity to maintain GI homeostasis.

The EP4 receptor is one of four known G-protein-coupled receptor subtypes for prostaglandin E_2_, which was a G_s_α-coupled and adenylyl cyclase stimulating receptor^43^. EP4 signaling plays a variety of roles through cAMP effectors^11^. However, abundant evidence from studies using pharmacological approaches and genetically modified mice suggests that EP4 can also be coupled to G_i_α, phosphatidylinositol3-kinase (PI3K), β-arr or β-catenin^44^. In our study, we found that EP4 receptor expression was downregulated in colitis, but increased after the treatment of PGE_2_ in DSS-induced experimental colitis, which contributed to the protection of PGE_2_. Our results showed that EP4 upregulated β-arr1, which protected the colon tissue. It has been shown that EP4 receptors play an important role in suppressing colitis and downregulating immune responses in DSS-induced colitis. Dey I *et al*.^30^ demonstrated that the role of EP4 signaling was different between early onset (pro-inflammatory on colonic epithelial cells) and late progressive stages of colitis (anti-inflammatory on immune cells in the lamina propria). Another study found that the EP4 agonist was effective in the treatment of IBD by enhancing epithelium survival and regeneration after DSS challenge^45^. However, it has been reported that the PGE_2_-EP4 system is the major PG system in preventing mucosal intestinal inflammation caused by DSS^46^. In our study, we demonstrated that human EP4 receptor expression was decreased in colitis. These results support the concept that EP4 contributes to ulcer healing.

β-arr1, which serves as a multifunctional adaptor and scaffold that mediates GPCRs, is expressed ubiquitously in mammalian tissues and mediates many signaling functions^15^. Moreover, it serves as a mediator for various E3 ubiquitin ligases and a transducer of numerous cellular functions, such as membrane-, cytosolic- and nuclear-associated signaling to regulate cellular responses to external stimuli^47, 48^. In addition to β-arr1 mediation of receptor internalization and degradation, previous studies have established that β-arr1 is involved in cell proliferation, apoptosis and differentiation by mediating PI3K/AKT signaling in response to a wide range of stimuli in different cell types^49^. It is now evident that β-arr1 is a critical cell signaling regulator in many inflammatory diseases, including sepsis, arthritis, autoimmune encephalomyelitis, and inflammatory bowel diseases^50–53^. However, the role of β-arr1 in intestinal epithelial cell apoptosis in colitis remains controversial. A recent study demonstrated that β-arr1 deficiency protected mice from experimental colitis^53^, while another study showed that β-arr1 in the non-hematopoietic compartment (likely epithelial cells) is protective during colitis^54^. The most recent studies have shown that β-arr1 exerts a protective effect in epithelial cells and stem cells^55, 56^. In our study, β-arr1 was found to be decreased in colonic specimens obtained from UC patients and in a mouse colitis model as determined by immunostaining and Western blotting. These findings further confirm that β-arr1 is involved in human and mice colitis and has a protective role. In our study, we found that deletion of *β-arr1* exacerbated DSS-induced colitis in mice. Furthermore, PGE_2_ protection was only observed in *β-arr1* WT mice. These findings demonstrated that β-arr1 plays a crucial role in the signaling process of cell proliferation.

Among the signaling pathways, PI3K/AKT signaling has been proposed to induce proliferative signals in IEC^57–59^. Previous reports have indicated that PI3K-mediated generation of PI-3, 4, 5-triphosphate recruits Akt to activate proliferation and survival signaling^59^. To investigate the involvement of PI3K signaling in colitis, activation of PI3K, p-Akt and Akt were assessed by western blot analysis. Immunohistochemical staining of PI3K and p-Akt found that β-arr1 activated PI3K/Akt signaling in colitis. To investigate the involvement of β-arr1-mediated PI3K signaling during PGE_2_-induced differentiation, activation of EP4, p-Akt and Akt were assessed by western blot analysis both *in vivo* and *in vitro*. We demonstrated that β-arr1 interacted with Akt, which provides a strong basis for PGE_2_/EP4 up-regulation of β-arr1 mediated Akt signaling. In conclusion, the present study revealed that PGE_2_ treatment helps to maintain colonic epithelial barrier integrity, and recovers normal expression and distribution of proteins by COX-1/PGE_2_/EP4 up-regulation of β-arr1/p-Akt signaling. Thus, these results suggest that PGE_2_ has a favorable therapeutic effect on DSS-induced ulcerative colitis, supporting its future development and clinical application in inflammatory bowel disease.

## Materials and Methods

### Ethics

All methods described in this study were performed in accordance with the approved guidelines by Research Ethics Committee of The Third Affiliated Hospital of Sun Yat-Sen University.

### Tissue samples

Frozen specimens from patients with UC and normal colon tissue samples were obtained from the Endoscopy Center of The Third Affiliated Hospital of Sun Yat-Sen University according to the ethical and legal standards. Our study was approved by the Research Ethics Committee of The Third Affiliated Hospital of Sun Yat-Sen University. Written informed consent was obtained from every patient prior to his/her inclusion in the study.

### Mice and treatment

All mice used in the animal experiments were approved by the Institutional Animal Care and Use Committee at the Sun Yat-Sen University. The experimental protocols in the present study, including all surgical procedures and animal usages, were performed according to the Guide for the Care and Use of Laboratory Animals by the National Institutes of Health (NIH) and approved by the Animal Care and Use Committee of Sun Yat-Sen University. The original *β-arr1* heterozygous mice in the C57BL/6 background were obtained from Dr. Robert J. Lefkowitz (Duke University Medical Center, Durham, NC, USA) as a generous gift. The mice were housed in micro-isolator cages containing wood shavings (3–6 mice per cage) and kept in a temperature-controlled (23 ± 5 °C) and humidity-controlled (50% ± 15%) environment with a 12:12 h light–dark cycle. Eight- to ten-week-old mice were used for all experiments. For colitis induction, mice were exposed to 5% DSS (MP Biomedical, LLC, Solon, OH) in their drinking water. Control mice were allowed to drink water without DDS concurrently. For indomethacin induction, mice were allowed to drink water with indomethacin (Sigma, St. Louis, MO, USA) at a dose of 4 mg/kg/day, which was administered to the animals during the entire experimental period. For PGE_2_ induction, mice received PGE_2_ (Cayman Chemical, Ann Arbor, MI) at a dose of 200 μg/150 μl/20 g body weight during DSS treatment.

### Determination of Disease Activity Index

Disease activity index scores were determined daily during the experiment, as previously described^60^. The extent of colitis, body weight, stool consistency and occult blood in the stool were monitored daily. Body weight was scored as follows: no weight loss was scored as 0; loss of 1–5% weight was registered as 1; loss of 6–10% weight was registered as 2; loss of 11–20% weight was registered as 3; and loss of greater than 20% weight was registered as 4. Stool consistency was scored as follows: 0 was scored for well-formed stool pellets, 2 was scored for pasty and semi-formed stools that did not adhere to the anus and 4 was scored for watery diarrhea that adhered to the anus. Bleeding, which was analyzed by the Hemoccult fecal occult blood test, was scored as follows: 0 was assigned for no blood, 2 was assigned for positive hemoccult, and 4 was assigned for gross bleeding. All of the scores were blindly confirmed by three independent individuals.

### Analysis of histology

Tissue sections (4 μm) of the colon were subjected to hematoxylin-eosin (H&E) for histological analysis. Histological scores were determined blindly based on previously described criteria^60^.

### Immunohistochemical and Immunofluorescence Staining

For immunohistochemical staining, 4 mm paraffin-embedded colon sections were deparaffinized in xylene and rehydrated in graded alcohol and then treated with 3% hydrogen peroxide, followed by antigen retrieval in boiling 0.1 M citrate (pH 6.0) buffer. Next, sections were then blocked with 10% goat serum (Zymed, Carlsbad, CA, USA) for 10 min and stained with antibodies directed against β-arr1 (kindly provided by Dr. Robert J Lefkowitz), EP4, Ki-67, and PCNA (Santa Cruz Biotechnology, Santa Cruz, CA, USA), and Cytokeratin-18 (Abcam, Cambridge, MA, USA), respectively. For immunofluorescence (IF) staining, the targeted protein was detected by the corresponding secondary antibody. Antibody-antigen complexes were visualized by incubation with biotin-conjugated secondary antibody and streptavidin Alexa 488 or 594 (Molecular Probes, China), and the nuclei were counterstained with 4′6-diamidino-2-phenylindole dihydrochloride (DAPI, Molecular Probes, Eugene, OR, USA). For double staining, a secondary targeted protein was detected after the initial protein-detection stage on the same slides. For cells IF staining, when the cells after the indicated treatment, they immediately fixed in 4% paraformaldehyde prior to the abovementioned procedure. The proliferative index was measured by quantifying a minimum of 20 randomly chosen fields following p-Akt (Cell Signaling Technology, Danvers, MA, USA) IF staining. The index was acquired by determining the number of p-Akt-positive cells against the total number of cells.

### Apoptosis Assays

TUNEL staining was performed using the ApopTag kit (Roche, Basel, Switzerland) according to the manufacturer’s instructions. Six mice from each group were examined. TUNEL-positive cells were counted in 100 randomly selected TUNEL-positive crypts as previously reported^60^.

### Real-time Polymerase Chain Reaction (Real-time PCR)

The mice were sacrificed after different treatments. We collected a piece of distal colon approximately 12 mm in length from the same location in all mice. Colonic mucosa was isolated by careful scraping and total RNA was isolated from the colon tissue using ISOGEN (Nippon Gene, Toyama, Japan). First strand cDNA synthesis was performed from 2 μg of total RNA using the Superscript Reverse Transcriptase (Toyobo Co., Ltd., Osaka, Japan) and random primers according to the manufacturer’s instructions in a final volume of 20 μl. Real-time RT-PCR was performed using the SYBR Greenmaster mixture (Thermo Fisher Scientific, USA) on an HT7500 system (Life Technologies, USA), and the reaction mixtures were incubated at 95 °C for 10 min, followed by 45 cycles of 95 °C for 15 s and 60 °C for 32 s. Melting curve analysis was performed to validate the generation of the expected PCR products. Each sample was analyzed in triplicate. The expression levels of each RNA were normalized to that of β-actin. The relative transcription expression of the mRNAs was calculated using the 2^−ΔΔCt^ method. The expression levels of *β-arr1*, *EP1*, *EP2*, *EP3*, *EP4*, *COX-1*, *COX-2* and *β-actin* in human or DSS-treated mice were determined by real-time PCR using specific primers. Primer sequences are listed in Supplementary Table 1.

### Enzyme-linked immunosorbent assay (ELISA)

The PGE_2_, PGD_2_, PGF_2_α and PGI_2_ concentration in biopsies of rectal mucosa were determined quantitatively using human and mice ELISA kits and performed in strict accordance with the manual of the experimental kit. Briefly, the ELISA kit (all from Cusabio Biotech Co., China) was equilibrated at room temperature for at least 30 min prior to preparation of the experimental solutions. A volume of 100 μl of standard solution was added into the reaction plate to produce the standard curve after the standard was dissolved. Next, a volume of 100 μl sample solution was added into each well, and the plate was incubated at 37 °C for 120 min. After washing the plate, 100 μl of freshly made working solution containing biotinylated antibodies was added into the wells and incubated at 37 °C for 60 min. After the second washing, 100 μl of freshly made solution containing horseradish peroxidase avidin was added and incubated at 37 °C for 60 min. The plate was then washed three times consecutively, and 100 μl of substrate solution was added and incubated in the dark at 37 °C for 15 to 30 min. Finally, stop solution was quickly added into the plate to terminate the reactions, and within 5 min. the optical density (OD) values were measured at a wavelength of 450 nm using a multifunction plate reader (Synergy 2; Bio Tek Instruments, Inc., Winooski, VT, USA). The standard curve was generated based on the measured OD values, and the levels of PGE_2_, PGD_2_, PGF_2α_ and PGI_2_ were calculated from the standard curve.

### Cell Culture and Transfection

The human colorectal cancer cell line HCT116 was routinely cultured in DMEM/F12 medium supplemented with 10% fetal bovine serum, 30 Uml^−1^ penicillin and 30 mg ml^−1^ streptomycin at 37 °C under 5% CO2. Small interfering RNA (siRNA) was performed according to the manufacturer’s instructions. The cells were transfected with 20 μM *β-arr1* using an RNA oligo kit (GenePharma, Shanghai, China) according to the manufacturer’s instructions, and the most effective sequence was selected to achieve a transfection efficiency of >90%. After incubation for 24 h, the transfection medium was replaced with regular culture medium before PGE_2_ administration.

### SDS-PAGE and Western Blotting

Proteins extracted from colonic mucosa and cell lysates were analyzed by SDS-PAGE. Separated proteins were transferred to nitrocellulose membranes, then blocked at room temperature for 1.5 hours in Tris- buffered saline with 0.1% Tween20 containing 5% skim milk and probed with primary antibodies at 4 °C overnight, and then, incubated with the appropriate peroxide-conjugated secondary antibody. Finally, images were acquired using ECL detection in a darkroom. The following antibodies were used: β-arr1 (kindly provided by Dr. Robert J Lefkowitz, Duke University Medical Center), p-Akt, Akt, PI3K (all from Cell Signaling Technology), EP4, COX-1, COX-2 (Santa Cruz) and β-actin (Sigma). The blots were detected with enhanced chemiluminescence (ECL) detection system (Amersham Pharmacia Biotech, Piscataway, NJ, USA) and exposed to X-ray film (Fuji Hyperfilm, Tokyo, Japan).The mean pixel density of the blots was analyzed by Quantity One softerware 4.6.2 (BioRad). β-actin was used as a loading control.

### Statistical Analysis

Statistical comparisons were made using SPSS20.0. All data are reported as means ± SD. To compare multiple groups, differences between groups were evaluated using parametric analysis of variance (ANOVA), followed by the Bonferroni’s post test. The differences between two groups were determined using Student’s t test. *P* < 0.05 was considered statistically significant.

## Electronic supplementary material

COX-1/PGE2/EP4 alleviates mucosal injury by upregulating β-arr1-mediated Akt signaling in colitis

## References

[1] Ordas I, Eckmann L, Talamini M, Baumgart DC, Sandborn WJ (2012). Ulcerative colitis. lancet..

[2] Molodecky, N. A. *et al*. Increasing incidence and prevalence of the inflammatory bowel diseases with time, based on systematic review. *Gastroenterology* **142**, 46–54, e30 (2012). 10.1053/j.gastro.2011.10.001 22001864

[3] Puspok A, Kiener HP, Oberhuber G (2000). Clinical, endoscopic, and histologic spectrum of nonsteroidal anti-Inflammatory drug-Induced lesions in the colon. Dis Colon Rectum..

[4] Smale S, Natt RS, Orchard TR, Russell AS, Bjarnason I (2001). Inflammatory bowel disease and spondylarthropathy. Arthritis Rheum..

[5] Bonner GF, Fakhri A, Vennamaneni SR (2004). A long-term cohort study of nonsteroidal anti-inflammatory drug use and disease activity in outpatients with inflammatory bowel disease. Inflamm Bowel Dis.

[6] Herschman HR (1994). Regulation of prostaglandin synthase-1 and prostaglandin synthase-2. Cancer Metastasis Rev..

[7] Cohn SM, Schloemann S, Tessner T, Seibert K, Stenson WF (1997). Crypt stem cell survival in the mouse intestinal epithelium is regulated by prostaglandins synthesized through cyclooxygenase-1. J Clin Invest..

[8] DuBois RN, Radhika A, Reddy BS, Entingh AJ (1996). Increased cyclooxygenase-2 levels in carcinogen-induced rat colonic tumors. Gastroenterology..

[9] Eberhart CE (1994). Up-regulation of cyclooxygenase 2 gene expression in human colorectal adenomas and adenocarcinomas. Gastroenterology..

[10] Singer II (1998). Cyclooxygenase 2 induced in colonic epithelial cells in inflammatory bowel disease. Gastroenterology..

[11] Narumiya S, Sugimoto Y, Ushikubi F (1999). Prostanoid receptors: structures, properties, and functions. Physiol Rev..

[12] Legler DF, Bruckner M, Uetz-von AE, Krause P (2010). Prostaglandin E2 at new glance: novel insights in functional diversity offer therapeutic chances. Int. J. Biochem. Cell Biol..

[13] Okuyama T (2002). Activation of prostaglandin E_2_-receptor EP2 and EP4 pathways induces growth inhibition in human gastric carcinoma cell lines. J Lab Clin Med..

[14] Buchanan FG (2006). Role of beta-arrestin 1 in the metastatic progression of colorectal cancer. Proc Natl Acad Sci USA.

[15] Shukla AK, Xiao K, Lefkowitz RJ (2011). Emerging paradigms of beta-arrestin-dependent seven transmembrane receptor signaling. Trends Biochem Sci..

[16] Wood H (2013). Alzheimer disease: Arrestin’ Alzheimer disease progression? beta-arrestin 2 is a potential therapeutic target. Nat Rev Neurol..

[17] Ohguro H (1993). Beta-arrestin and arrestin are recognized by autoantibodies in sera from multiple sclerosis patients. Proc Natl Acad Sci USA.

[18] Zeng LX (2014). β-Arrestin2 encourages inflammation-induced epithelial apoptosis through ER stress/PUMA in colitis. Mucosal Immunol..

[19] Chen T (2015). Insulin-like growth factor-1 contributes to mucosal repair by β-Arrestin2–mediated extracellular signal-related kinase signaling in experimental colitis. Am J Pathol..

[20] Leduc M (2009). Functional selectivity of natural and synthetic prostaglandin EP4 receptor ligands. J Pharmacol Exp Ther..

[21] Schulte G, Shenoy SK (2011). β-Arrestin and dishevelled coordinate biased signaling. Proc Natl Acad Sci USA.

[22] Hu S (2013). Involvement of β-arrestins in cancer progression. Mol Biol Rep..

[23] Kawahara K, Hohjoh H, Inazumi T, Tsuchiya S, Sugimoto Y (2015). Prostaglandin E_2_-induced inflammation: relevance of prostaglandin E receptors. Biochim Biophys Acta..

[24] Halter F, Tarnawski AS, Schmassmann A, Peskar BM (2001). Cyclooxygenase 2-implications on maintenance of gastric mucosal integrity and ulcer healing: controversial issues and perspectives. Gut..

[25] Stenson WF (2007). Prostaglandins and epithelial response to injury. Curr Opin Gastroenterol..

[26] Tessner TG, Cohn SM, Schloemann S, Stenson WF (1998). Prostaglandins prevent decreased epithelial cell proliferation associated with dextran sodium sulfate injury in mice. Gastroenterology..

[27] Houchen CW, Stenson WF, Cohn SM (2000). Disruption of cyclooxygenase-1 gene results in an impaired response to radiation injury. Am J Physiol Gastrointest Liver Physiol..

[28] Wiercińska-Drapało A, Flisiak R, Prokopowicz D (2001). Plasma and mucosal prostaglandin E_2_ as a surrogate marker of ulcerative colitis activity. Rocz Akad Med Bialymst..

[29] Wiercińska-Drapa OA, Flisiak R, Prokopowicz D (1999). Effects of ulcerative colitis activity on plasma and mucosal prostaglandin E_2_ concentration. Prostagothlipidm..

[30] Dey I, Lejeune M, Chadee K (2006). Prostaglandin E_2_ receptor distribution and function in the gastrointestinal tract. Br J Pharmacol..

[31] Casellas F (1994). Intraluminal colonic release of immunoreactive tumor necrosis factor in chronic ulcerative colitis. Clin Sci.

[32] Dai L (2015). Inverse Expression of prostaglandin E_2_-related enzymes highlights differences between diverticulitis and inflammatory bowel disease. Dig Dis Sci..

[33] Miyoshi H (2017). Prostaglandin E_2_ promotes intestinal repair through an adaptive cellular response of the epithelium. EMBO J..

[34] Ferrer R, Moreno JJ (2010). Role of eicosanoids on intestinal epithelial homeostasis. Biochem Pharmacol..

[35] Tanaka K (2009). Inhibition of both COX-1 and COX-2 and resulting decrease in the level of prostaglandins E_2_ is responsible for non-steroidal anti-inflammatory drug (NSAID)-dependent exacerbation of colitis. Eur J Pharmacol..

[36] Melgar S, Drmotova M, Rehnstrom E, Jansson L, Michaelsson E (2006). Local production of chemokines and prostaglandin E_2_ in the acute, chronic and recovery phase of murine experimental colitis. Cytokine..

[37] Yamashita S (1993). Studies on changes of colonic mucosal PGE_2_ levels and tissue localization in experimental colitis. Gastroenterol Jpn..

[38] Zifroni A, Treves AJ, Sachar DB, Rachmilewitz D (1983). Prostanoid synthesis by cultured intestinal epithelial and mononuclear cells in inflammatory bowel disease. Gut..

[39] Jostins L (2012). Host-microbe interactions have shaped the genetic architecture of inflammatory bowel disease. Nature..

[40] Zhang Y (2015). Tissue regeneration. Inhibition of the prostaglandin-degrading enzyme 15-PGDH potentiates tissue regeneration. Science..

[41] Kandil HM, Argenzio RA, Sartor RB (2000). Low endogenous prostaglandin E_2_ predisposes to relapsing inflammation in experimental rat enterocolitis. Dig Dis Sci..

[42] Hara S (2010). Prostaglandin E synthases: understanding their pathophysiological roles through mouse genetic models. Biochimie..

[43] Fujino H, Regan JW (2006). EP(4) prostanoid receptor coupling to a pertussis toxin-sensitive inhibitory G protein. Mol Pharmacol..

[44] Yokoyama U, Iwatsubo K, Umemura M, Fujita T, Ishikawa Y (2013). The prostanoid EP4 receptor and its signaling pathway. Pharmacol Rev..

[45] Jiang GL (2007). The prevention of colitis by E prostanoid receptor 4 agonist through enhancement of epithelium survival and regeneration. J Pharmacol Exp Ther..

[46] Zimecki M (2012). Potential therapeutic interventions via EP2/EP4 prostaglandin receptors. Postepy Hig Med Dosw (Online)..

[47] Shukla AK (2014). Visualization of arrestin recruitment by a G-protein-coupled receptor. Nature..

[48] Shenoy SK (2014). Arrestin interaction with E3 ubiquitin ligases and deubiquitinases: functional and therapeutic implications. Handb Exp Pharmacol..

[49] Kovacs JJ, Hara MR, Davenport CL, Kim J, Lefkowitz RJ (2009). Arrestin development: emerging roles for beta-arrestins in developmental signaling pathways. Dev. Cell..

[50] Sharma D, Malik A, Lee E, Britton RA, Parameswaran N (2013). Gene dosage-dependent negative regulatory role of beta-arrestin-2 in polymicrobial infection-induced inflammation. Infect Immun..

[51] Li J (2013). Deficiency of beta-arrestin1 ameliorates collagen-induced arthritis with impaired TH17 Cell differentiation. Proc Natl Acad Sci USA.

[52] Shi Y (2007). Critical regulation of CD4^+^ T Cell survival and autoimmunity by beta-arrestin 1. Nat Immunol..

[53] Lee T (2013). Beta-arrestin-1 deficiency protects mice from experimental colitis. Am J Pathol..

[54] Lee T, Lee E, Arrollo D, Lucas PC, Parameswaran N (2016). Non-hematopoietic beta-arrestin1 confers protection against experimental colitis. J Cell Physiol..

[55] Tan S (2015). β-arrestin-1 protects against endoplasmic reticulum stress/p53-upregulated modulator of apoptosis-mediated apoptosis via repressing p-p65/inducible nitric oxide synthase in portal hypertensive gastropathy. Free Radical Bio Med..

[56] Zhan Y (2016). β-Arrestin1 inhibits chemotherapy-induced intestinal stem cell apoptosis and mucositis. Cell Death Dis.

[57] Sheng H, Shao J, Townsend CJ, Evers BM (2003). Phosphatidylinositol 3-kinase mediates proliferative signals in intestinal epithelial cells. Gut..

[58] He XC (2007). PTEN-deficient intestinal stem cells initiate intestinal polyposis. Nat Genet..

[59] Engelman JA, Luo J, Cantley LC (2006). The evolution of phosphatidylinositol 3-kinases as regulators of growth and metabolism. Nat Rev Genet..

[60] Qiu W (2011). PUMA-mediated intestinal epithelial apoptosis contributes to ulcerative colitis in humans and mice. J Clin Invest..

