# Supplementary material for: COX-1/PGE2/EP4 alleviates mucosal injury by upregulating β-arr1-mediated Akt signaling in colitis
Source: Sci Rep. 2017 Apr 21;7:1055. doi: 10.1038/s41598-017-01169-6 (PMC5430694; doi:10.1038/s41598-017-01169-6)
Supplement: Supplementary file 1 — COX-1/PGE2/EP4 alleviates mucosal injury by upregulating β-arr1-mediated Akt signaling in colitis [file 41598_2017_1169_MOESM1_ESM.pdf]

Supplementary Information

COX-1/PGE_2/EP4 alleviates mucosal injury by upregulating β-arr1-mediated Akt signaling in colitis

Running Title: COX-1/PGE_2/EP4 alleviates mucosal injury in colitis

Xiaojie Peng[1,2], Jianzhong Li[1,2], Siwei Tan[1], Minyi Xu[1], Jin Tao[1], Jie Jiang[1], Huiling Liu[1]&Bin Wu*,[1]

[1]Department of Gastroenterology, The Third Affiliated Hospital of Sun Yat-Sen University, Guangzhou, China.

[2]These authors contributed equally to this work.

[*]**Corresponding author:** Bin Wu, M.D. & Ph.D., Department of Gastroenterology, The Third Affiliated Hospital of Sun Yat-Sen University, No. 600 Tianhe Road, Guangzhou 510630, China. Tel: +86-20-85252801; Fax: +86-20-85253336; Email: wubin6@mail.sysu.edu.cn.

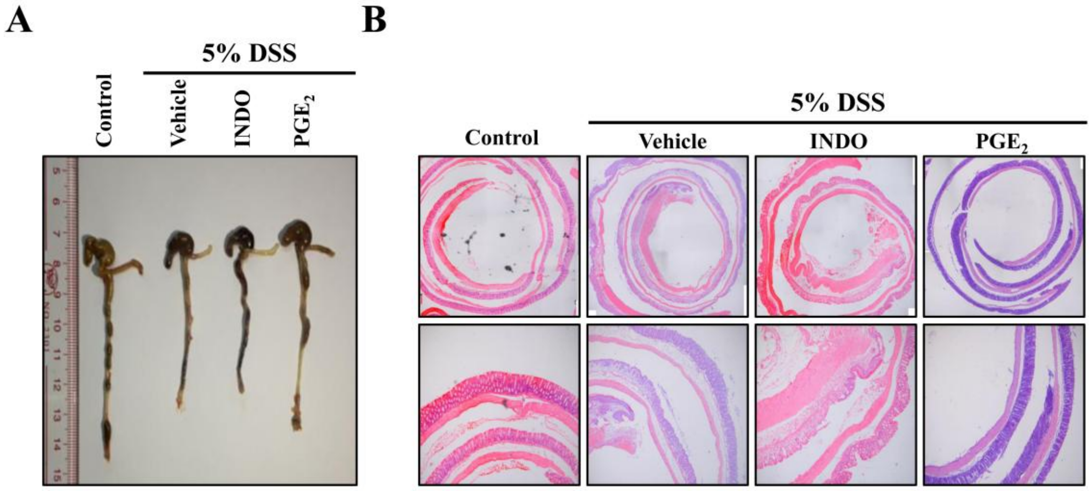

Supplementary Fig.S1. PGE_2/EP4 alleviates mucosal injury in colitis. (**A**) Colon length of control group , 5% DSS treatment group, INDO treatment group and PGE_2 treatment group mice. (**B**) Colon histopathology of control group, DSS treatment group , INDO treatment group and PGE_2 treatment group mice.

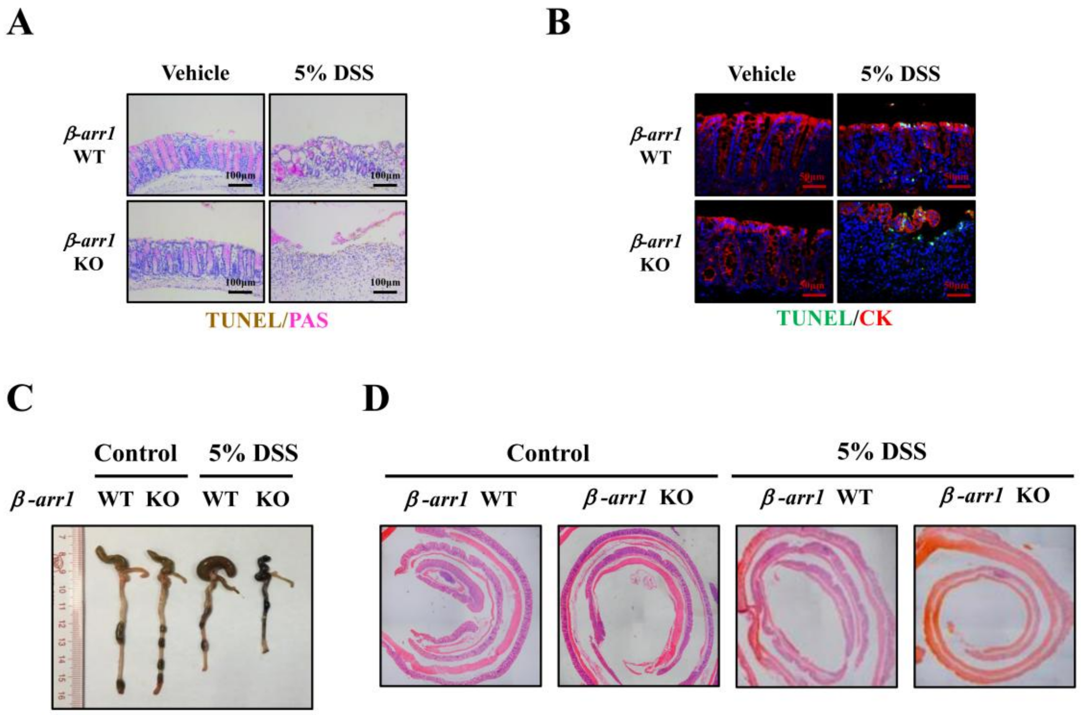

Supplementary Fig.S2. Targeted deletion of *β*-arr1 exacerbates DSS induced colitis in mice. (**A**).Double stain for PAS and TUNEL in colonic sections of *β-arr1*WT and KO littermates mice at indicated time points (×200). PAS for goblet cells is pink (×200). (**B**) Double immunofluorescence stain for cytokeratin and TUNEL at indicated group (400×). Nuclei were stained with DAPI in blue. TUNEL were visualized in green and cytokeratin was stained in red. The merging positive signals of TUNEL and cytokeratin were visualized in yellow. (**C**) Colon length of *β*-arr1WT and KO mice in control group and 5% DSS treatment group. Data pooled from 3 independent experiments, n = 6 . (**D**) Colon histopathology of *β*-arr1WT and KO mice in control group and 5% DSS treatment group. Data pooled from 3 independent experiments, n = 6 .

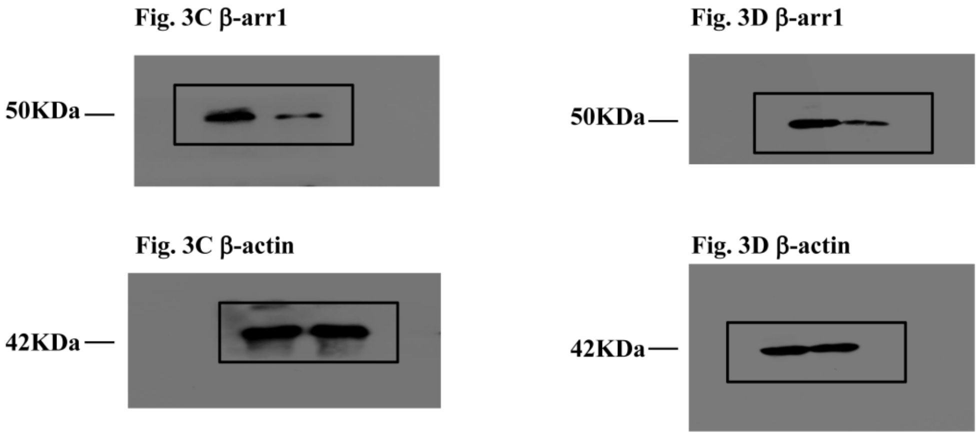

Supplementary Figure S3. Uncropped gel images with size marker indications. Sold lines are cropping lines.

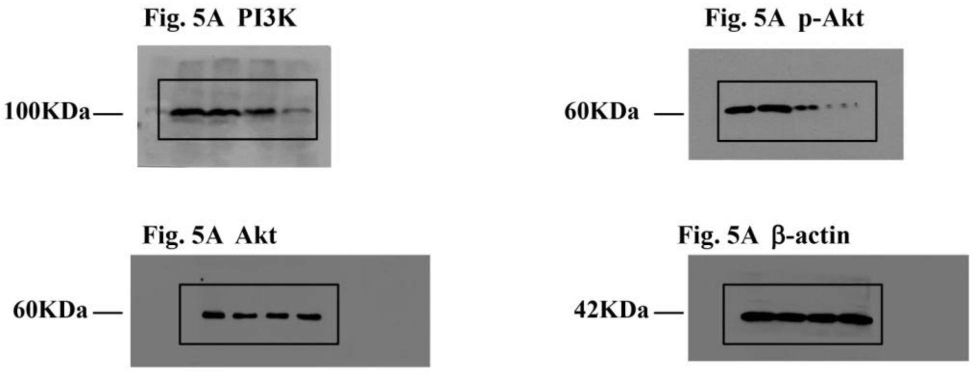

Supplementary Figure S4. Uncropped gel images with size marker indications. Sold lines are cropping lines.

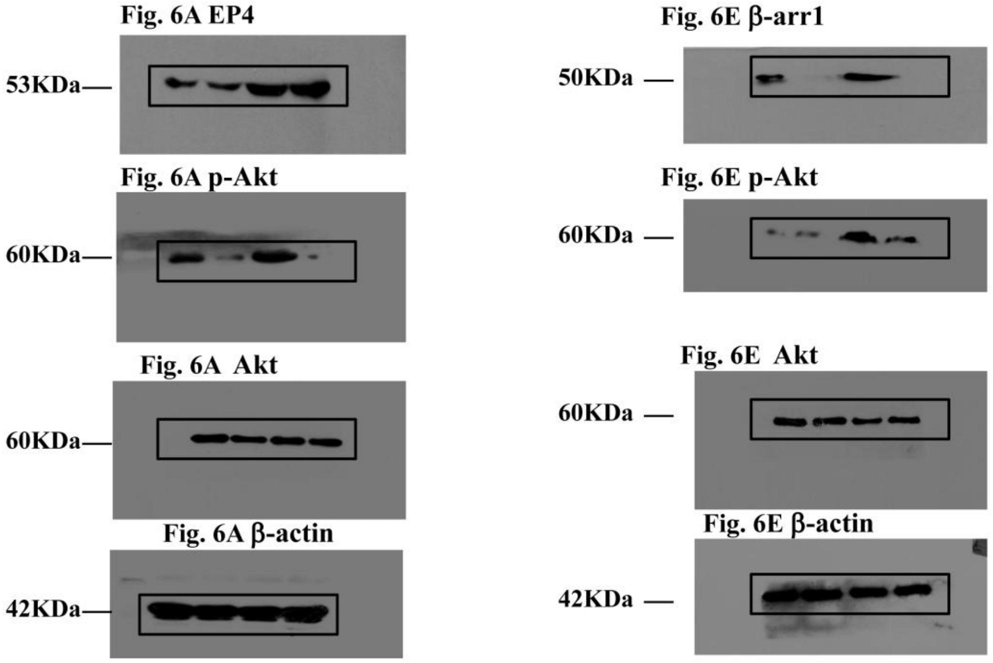

Supplementary Figure S5. Uncropped gel images with size marker indications. Sold lines are cropping lines.

Table S1. Sequences of the amplification primers used in this study

Sequence name			Sequence
Human	*β-arr1*	forward	5’-GCGAGCACGCTTACCCTTT-3’,
		reverse	5’- CAAGCCTTCCCCGTGTCTTC-3’;
Human	*EP1*	forward	5’-AGCTTGTCGGTATCATGGTGG-3’,
		reverse	5’-AAGAGGCGAAGCAGTTGGC-3’;
Human	*EP2*	forward	5’-GACTAATGCGTTCAGTCCTCTG-3’,
		reverse	5’-GGTCAGCCTGTTTACTGGCA-3’;
Human	*EP3*	forward	5’-GTCGTCATCGTCGTGTACCTG-3,
		reverse	5-AGTCATGGTCAGCCCGAAAAA-3’,
Human	*EP4*	forward	5’-CATCATCTGCGCCATGAGTGT-3’,
		reverse	5’-GCTTGTCCACGTAGTGGCT-3’;
Human	*β-actin*	forward	5′-GTCTTCCCCTCCATCGTG-3′,
		reverse	5′- AGGGTGAGGATGCCTCTCTT-3′,
Human	*COX-1*	forward	5′- TGCGCTCCAACCTTATCCC-3′,
		reverse	5′- AGAGGGCAGAATACGAGTGTAA-3′,
Human	*COX-2*	forward	5′- CTGGCGCTCAGCCATACAG-3′,
		reverse	5′- CGCACTTATACTGGTCAAATCCC-3′,
Mouse	*β-arr1*	forward	5’-TGGGCGACAAAGGGACACGA-3’,
		reverse	5’-AACAGGTCTTTCCGAAAAGTCG-3’;
Mouse	*EP1*	forward	5’- GGGCTTAACCTGAGCCTAGC -3’,
		reverse	5’-GTGATGTGCCATTATCGCCTG-3’;
Mouse	*EP2*	forward	5’-TCCCTAAAGGAAAAGTGGGACC-3’,
		reverse	5’-GAGCGCATTAACCTCAGGACC-3’.
Mouse	*EP3*	forward	5’-CCGGAGCACTCTGCTGAAG-3’,
		reverse	5’- CCCCACTAAGTCGGTGAGC-3’.
Mouse	*EP4*	forward	5’-ACCATTCCTAGATCGAACCGT-3’,
		reverse	5’-CACCACCCCGAAGATGAACAT-3’.
Mouse	*β-actin*	forward	5’-GGCTGTATTCCCCTCCATCG-3’,
		reverse	5’- CCAGTTGGTAACAATGCCATGT-3’.
Mouse	*COX-1*	forward	5’- ATGAGTCGAAGGAGTCTCTCG-3’,
		reverse	5’- GCACGGATAGTAACAACAGGGA-3’.
Mouse	*COX-2*	forward	5’- TGAGCAACTATTCCAAACCAGC-3’,
		reverse	5’- GCACGTAGTCTTCGATCACTATC-3’.
